# Potential Small Guide RNAs for tRNase Z^L^ from Human Plasma, Peripheral Blood Mononuclear Cells, and Cultured Cell Lines

**DOI:** 10.1371/journal.pone.0118631

**Published:** 2015-03-02

**Authors:** Sho Ninomiya, Mitsuoki Kawano, Takashi Abe, Tatsuya Ishikawa, Masayuki Takahashi, Masato Tamura, Yoshiaki Takahashi, Masayuki Nashimoto

**Affiliations:** 1 Department of Applied Life Sciences, Niigata University of Pharmacy and Applied Life Sciences, Niigata, Japan; 2 Department of Biochemistry and Molecular Biology, Hokkaido University Graduate School of Dental Medicine, Sapporo, Japan; 3 Department of Information Engineering, Niigata University, Niigata, Japan; 4 Laboratory of Biochemistry, Niigata University Graduate School of Health Sciences, Niigata, Japan; 5 Research Institute for Healthy Living, Niigata University of Pharmacy and Applied Life Sciences, Niigata, Japan; Faculdade de Medicina Dentária, Universidade do Porto, PORTUGAL

## Abstract

Several pieces of evidence suggest that small RNA degradation products together with tRNase Z^L^ appear to form another layer of the whole gene regulatory network. The degraded RNA such as a 5′-half-tRNA and an rRNA fragment function as small guide RNA (sgRNA) to guide the enzyme to target RNA. We were curious whether there exist RNAs in plasma that can function as sgRNAs for tRNase Z^L^, whether these RNAs are working as signaling molecules between cells to fulfill physiological roles, and whether there are any differences in plasma sgRNA species and levels between normal and pathological conditions. Here, we analyzed small plasma RNAs from three healthy persons and three multiple myeloma patients for potential sgRNAs by deep sequencing. We also examined small RNAs from peripheral blood mononuclear cells (PBMC) of three healthy persons and three myeloma patients and from various cultured human cell lines for sgRNAs. We found that read-number distribution patterns of plasma and PBMC RNAs differ between persons in the range of 5–40 nt and that there are many RNA species that exist significantly more or less abundantly in the plasma or PBMC of the myeloma patients than those of the healthy persons. Furthermore, we found that there are many potential sgRNAs in the 5–40-nt RNAs and that, among them, a 31-nt RNA fragment derived from 94-nt Y4-RNA, which can function as a 5′-half-tRNA-type sgRNA, is overwhelmingly abundant in the plasma of 2/3 of the examinees. These observations suggest that the gene regulatory network via tRNase Z^L^ and sgRNA may be extended intercellularly.

## Introduction

Sometimes great discoveries come from apparently meaningless noises as exemplified by the discovery of the cosmic background radiation [1,2]. Small RNA degradation products may be such noises, which could expand the extant RNA world [3,4]. Our first encounter with such RNA was with 3′-truncated tRNA, which was found as an RNA cofactor of a sequence-specific endoribonuclease activity in mouse cell extracts [5]. Although we have shown that the 3′-truncated tRNA can form a complex with the tRNA processing endoribonuclease tRNase Z^L^ and guide the enzyme to artificial target RNA molecules via four base-pairings [5–8], it remains to be elucidated whether this degraded tRNA has a physiological role or not.

**Fig 1:**
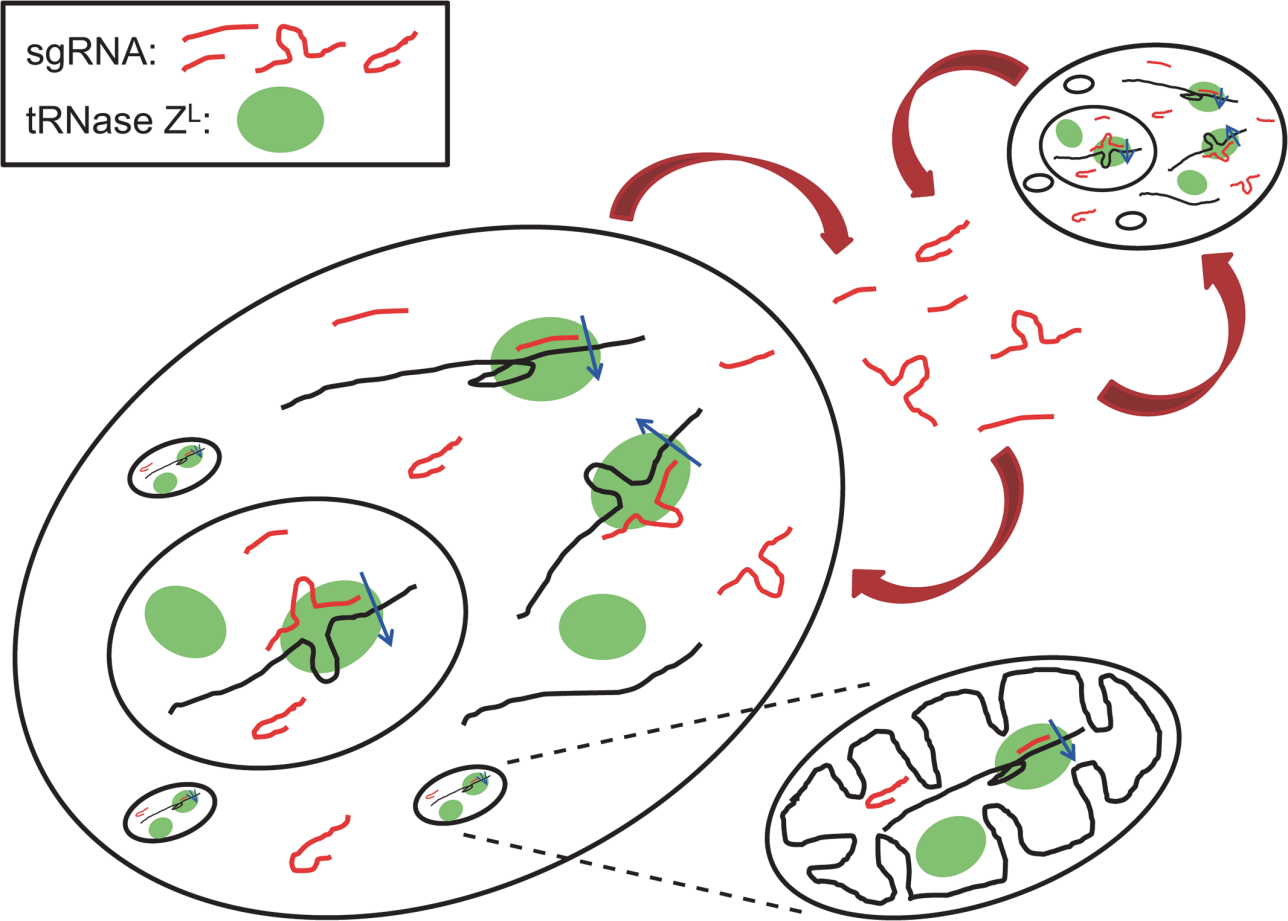
Schematic of a possible gene regulatory network via sgRNA and tRNase Z^L^. Small RNA degradation products from tRNA or rRNA can work as sgRNA for tRNase Z^L^, which exists ubiquitously in cells including mitochondria [9]. These RNAs may be also working as signaling molecules between cells.

After an ∼20-year interval, we and other groups have discovered many other forms of tRNA degradation products, a subset of which have been suggested to have physiological functions [9–11]. We have found various 5′- and 3′-half-tRNAs in HEK293 cells that co-immunoprecipitate with tRNase Z^L^, and have shown that one of them, a 5′-half-tRNA^Glu^, can function as a guide RNA for tRNase Z^L^ to cleave a target RNA *in vitro* [9]. The cellular PPM1F mRNA appears to be downregulated by tRNase Z^L^ guided by the 5′-half-tRNA^Glu^, and the HDAC4 mRNA is indirectly upregulated by increasing tRNase Z^L^ and/or 5′-half-tRNA^Glu^ levels. Furthermore, we have shown that various RNA fragments derived from rRNAs and snRNAs also co-immunoprecipitate with tRNase Z^L^ and that the DYNC1H1 mRNA appears to be downregulated by tRNase Z^L^ guided by a 28S rRNA 3′-terminal fragment [9]. These observations suggest that small RNA degradation products together with tRNase Z^L^ appear to form another layer of the whole gene regulatory network (Fig. 1).

The existence of this layer of the network would be supported by the property of tRNase Z^L^ that it can cleave any target RNA at any desired site under the direction of a small guide RNA (sgRNA) by recognizing a pre-tRNA-like or micro-pre-tRNA-like complex formed between the target RNA and the sgRNA [12–18]. Based on this enzymatic property, we have been developing an sgRNA-mediated gene silencing technology termed tRNase Z^L^-utilizing efficacious gene silencing (TRUE gene silencing) [19–24]. The sgRNA is usually 7–31 nt and categorized into four groups, 5′-half-tRNA, 14-nt linear RNA, heptamer RNA, and hook RNA, but 5- and 6-nt RNAs and >31-nt RNAs can also be sgRNA [12–18]. Although the efficacy of TRUE gene silencing differs depending on target RNAs, cell types, and sgRNA types, on the whole, downregulation levels of target RNAs are moderate. Nevertheless, in the cases of heptamer-type sgRNAs targeting BCL2 and WT1 mRNAs, they can efficiently induce apoptosis in human leukemia cells in spite of weak downregulation of the target mRNAs, suggesting the presence of additional RNA targets and/or additional mechanisms [25,26].

We have shown that naked sgRNAs can be taken up by living cells without any transfection reagents and that they can downregulate their target RNA levels and/or induce apoptosis [24–27]. This observation inspired us to investigate whether there exist RNAs in plasma that can function as sgRNAs for tRNase Z^L^, whether these RNAs are working as signaling molecules between cells to fulfill physiological roles, and whether there are any differences in plasma sgRNA species and levels between normal and pathological conditions. In this paper, we analyzed small plasma RNAs from three healthy persons and three multiple myeloma patients for potential sgRNAs using a next-generation sequencer. We also examined small RNAs from peripheral blood mononuclear cells (PBMC) of three healthy persons and three myeloma patients and from various cultured human cell lines for sgRNAs. We found that read-number distribution patterns of plasma and PBMC RNAs differ between persons in the range of 5–40 nt and that there are many RNA species that exist significantly more or less abundantly in the plasma or PBMC of the myeloma patients than those of the healthy persons. Furthermore, we found that there are many potential sgRNAs in the 5–40-nt RNAs and that, among them, a 31-nt RNA fragment derived from 94-nt Y4-RNA, which can function as a 5′-half-tRNA-type sgRNA, is overwhelmingly abundant in the plasma of 2/3 of the examinees.

## Materials and Methods

### Cell culture

Human cell lines, Jurkat [28], KMM-1 [29], HL60 [30], RPMI-8226 [31], and DAUDI (obtained from RIKEN BioResource Center, Tsukuba, Japan), were cultured in RPMI-1640 (Wako, Osaka, Japan) supplemented with 10% fetal bovine serum (MP Biomedicals Japan, Tokyo, Japan) and 1% penicillin-streptomycin (Invitrogen Japan, Tokyo, Japan) at 37°C in 5% CO_2_ humidified incubator. Likewise, A549 [32], HepG2 [33], HeLa [34], HEK293 [35], Huh-7 [36], and IMR-90 [37] cells were cultured in DME media (Wako, Osaka, Japan). Cultured human cells were harvested at ∼2 × 10^5^ cells/ml.

### RNA preparation

Total RNA samples from PBMC of three healthy persons (N4–N6) and three multiple myeloma patients (MM4–MM6) were purchased from AllCells (California, USA). And plasma from three healthy persons (N1–N3) and three myeloma patients (MM1–MM3) were also obtained from AllCells. Written informed consent was obtained from each person by AllCells. In the case that human samples to be analyzed are purchased from a company which obtained written informed consent from their origin persons, approval from the ethics committee is not needed in Niigata University of Pharmacy and Applied Life Sciences. The information about these persons is presented in S1 Table, and the examinees are different persons with the exception that MM1 and MM6 are the identical person. Total plasma RNA was prepared using a QIAamp Circulating Nucleic Acid Kit (Qiagen Japan, Tokyo, Japan). Total RNA of cultured human cells was extracted with RNAiso Plus (Takara Bio, Otsu, Japan) by the acid guanidinium-phenol-chloroform method [38].

### cDNA synthesis and deep sequencing

A cDNA library was constructed from above each total RNA (1.2–2.0 μg) basically as described before [39]. The 3′ pre-adenylated DNA adapter 5′-pATCTCGTATGCCGTCTTCTGCTTGT-3′ (in which the 3′-end thymidine is bound in the 3′-to-3′ direction) and one of the six 5′ RNA adapters were added to the 3′-end and the 5′-end, respectively, of each RNA molecule. The sequences of the 5′-adapters containing a barcode (denoted by an underline) are as follows: 5′-guucagaguucuacaguccgacgaucgaaa-3′, 5′-guucagaguucuacaguccgacgaucaaaa-3′, 5′-guucagaguucuacaguccgacgaucagaa-3′, 5′-guucagaguucuacaguccgacgaucacaa-3′, 5′-guucagaguucuacaguccgacgauccaaa-3′, and 5′-guucagaguucuacaguccgacgaucUaaa-3′. The RNA molecules with both adapters were reverse-transcribed with the DNA primer 5′-CAAGCAGAAGACGGCATACGA-3′ in the presence of the 22-nt locked-nucleic-acid-modified DNA Dimer-Eliminator-22 [39], and the resultant cDNA molecules were amplified by 14 or 18 (only for plasma RNA samples) cycles of the polymerase chain reaction (PCR) with the primer pair 5′-AATGATACGGCGACCACCGACAGGTTCAGAGTTCTACAGTCCGA-3′ and 5′-CAAGCAGAAGACGGCATACGA-3′. The amplified cDNAs were separated by polyacrylamide gel electrophoresis, and ∼80–120-base-pair cDNAs, which correspond to ∼5–45-nt original RNAs, were recovered and purified. The cDNA libraries from 23 RNA samples were listed with an RNA origin and a barcode sequence in S2 Table.

The purified cDNA samples were subjected to the next-generation sequencer Illumina HiSeq 2000. A maximum of six cDNA libraries tagged with the different barcodes were run at the same time. These sequence analyses were carried out by RIKEN GeNAS (Yokohama, Japan).

### Bioinformatics and statistical analysis

After base calling, we removed linker sequences and artificial linker adapter sequences using TagDust v1.13 [40], and identified the reads matching the ribosomal DNA repeating unit (GenBank accession number U13369) within two mismatches. Only the remaining reads were mapped to the human genome assembly (GRCh37/hg19) using BWA v0.5.9 [41]. Relative frequencies of 10 RNA categories, mRNA, rRNA, scRNA, snRNA, srpRNA, miRNA, lncRNA, piRNA, snoRNA, and tRNA, were calculated based on the reads mapped within these RNA gene regions. The RNA annotations were carried out based on mRNA and tRNA sequences from the UCSC Known Genes database (http://genome.ucsc.edu/), ncRNA sequences from National Center for Biotechnology Information (http://www.ncbi.nlm.nih.gov/), piRNA sequences from Girard et al. [42], miRNA sequences from miRBase [43], and the remaining RNAs’ sequences from RepeatMasker (http://repeatmasker.org/). Since various sequences that closely match a part of the 5′-adapter sequences were present in the read sequences from plasma of the three myeloma patients, these sequences were further removed for frequency distribution analysis and abundant RNA sequence analysis.

The statistical significance of differences between groups was evaluated by the two-tailed Student’s *t*-test (http://studentsttest.com/). The differences with a *P* value <0.05 were regarded as statistically significant. The binomial test was carried out using the Microsoft Excel 2010.

### Accession number

Small RNA sequences were deposited in the DNA Data Bank of Japan (http://www.ddbj.nig.ac.jp), accessions DRA002550, DRX021324–DRX021346, and DRR023270–DRR023292.

### *In vitro* RNA cleavage assay

A 31-nt Y4-RNA fragment 5′-GGCUGGUCCGAUGGUAGUGGGUUAUCAGAAC-3′ with 5′- and 3′-phosphates and full 2′-*O*-methyl modifications was chemically synthesized by Nippon Bioservice (Saitama, Japan). A 46-nt target RNA, RNA-Y 5′-GGAGUUCUAGAUUCCAGGUUCGACUCCUGGACCAGCCGGUGUAAGC-3′, was synthesized with T7 RNA polymerase from a corresponding synthetic DNA template basically as described before [12]. The RNA transcript for RNA-Y was subsequently 5′-labeled with fluorescein as described before [18].

*In vitro* RNA cleavage assays for the fluorescein-5′-labeled RNA-Y (2 pmol) were carried out at the optimum temperature 50°C in the presence of the unlabeled 31-nt Y4-RNA fragment (20 pmol) using histidine-tagged human Δ30 tRNase Z^L^ (50 ng) in a mixture (6 μl) containing 10 mM Tris-HCl (pH 7.5) and 10 mM MgCl_2_ [9]. After resolution of the reaction products on a 10% polyacrylamide-8 M urea gel, the gel was analyzed with a Typhoon 9210 (GE Healthcare Japan, Tokyo, Japan).

## Results and Discussion

### Small RNA from human plasma

**Fig 2:**
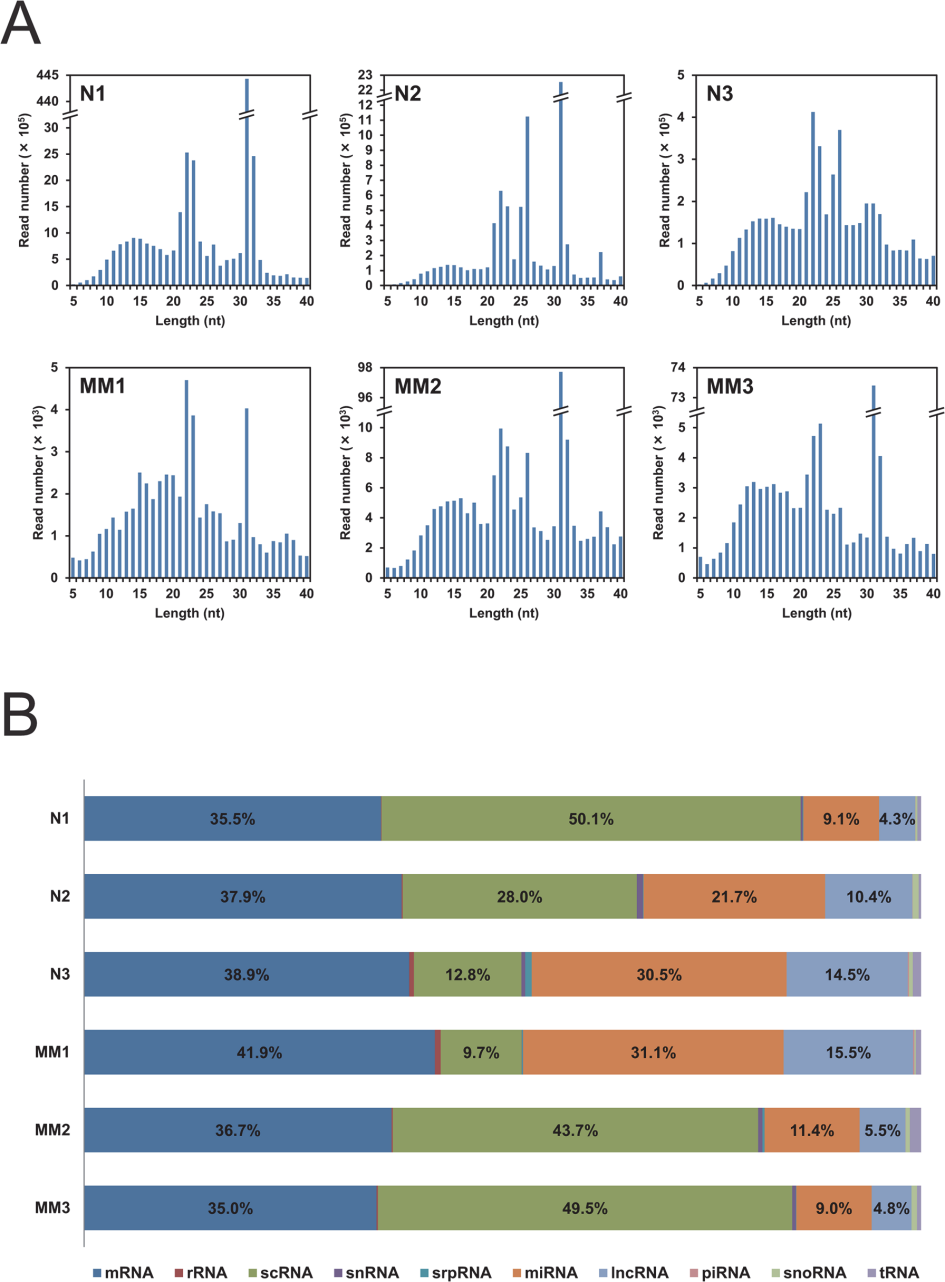
Deep sequencing analysis of small RNA from human plasma. (A) Frequencies of 5–40-nt RNAs. Read numbers of 5–40-nt plasma RNAs from three healthy persons (N1–N3) and three myeloma patients (MM1–MM3) are presented. (B) Relative frequencies of the 10 RNA categories. Each read sequence was assigned to the categories mRNA, rRNA, scRNA, snRNA, srpRNA, miRNA, lncRNA, piRNA, snoRNA, and/or tRNA, and their relative frequencies are shown for the above six persons.

We analyzed small plasma RNAs from three healthy persons (N1–N3) and three multiple myeloma patients (MM1–MM3) for their frequencies and sequences by deep sequencing. The frequencies of 5–40-nt plasma RNAs are graphed for each person (Fig. 2A). Although there were common peaks in the frequency distributions, each person showed his/her unique distribution pattern. Each distribution had peaks at 14/15/16, 22/23, 26, 31, and 37 nt in common. Notably, frequencies of the 31-nt RNA were highest in the distributions from two healthy persons and two myeloma patients, and those in three persons, N1, MM2, and MM3, were overwhelmingly high.

Each read RNA sequence was assigned to its potential original transcript(s), which are grouped into the 10 RNA categories mRNA, rRNA, scRNA, snRNA, srpRNA, miRNA, lncRNA, piRNA, snoRNA, and/or tRNA, and relative frequencies of these RNA categories are shown for each person in Fig. 2B. Although the categories mRNA, scRNA, miRNA, and lncRNA occupied more than 97% in each person, the relative frequency distributions differed from person to person. The percentages of the category mRNA were relatively constant and ranged between 35 and 42%, while those of the categories scRNA, miRNA, and lncRNA changed largely by 10–50%, 9–31%, and 4–15%, respectively. We found no obvious features in the frequency distributions that distinguish the myeloma persons from the healthy persons.

**Fig 3:**
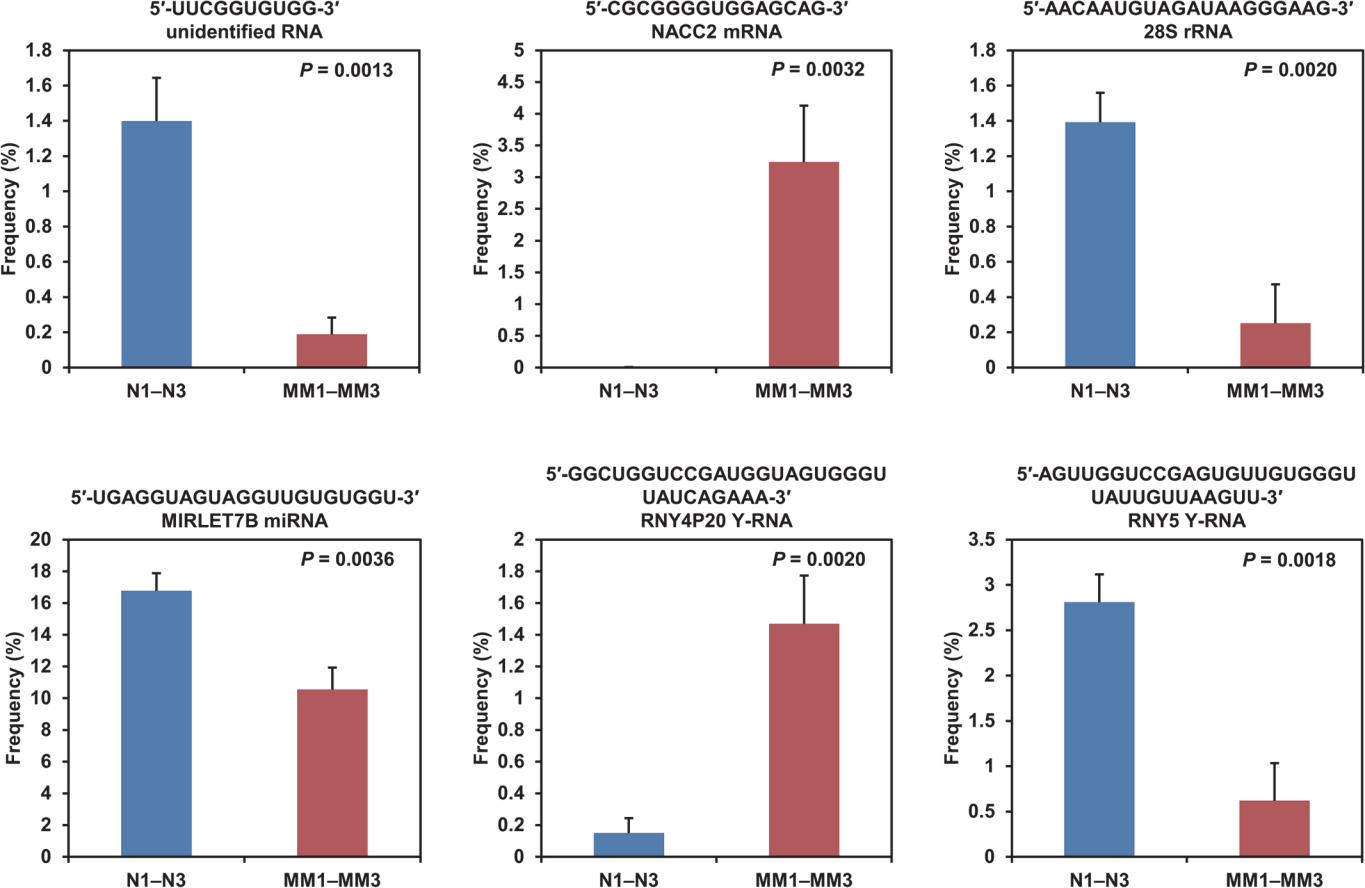
Analysis of RNA species abundant in human plasma. Average frequencies of each of the six representative RNA species in plasma from three myeloma patients (MM1–MM3) and in that from three healthy persons (N1–N3) are graphed. The vertical axis represents the percentage of the read number of each RNA species to the total read number of RNAs with the same nucleotide length. Sequences of the RNA species are presented with their potential original transcript name. Error bars denote standard deviations. These RNA species were significantly more or less abundant in the plasma from the myeloma patients.

Next we listed top 5 abundant RNA sequences for each of 5 to 40 nucleotides in each person and compared frequencies of each RNA sequence among the 6 persons in order to find small RNA species whose frequencies distinguish the myeloma patients from the healthy persons. There were 753 comparing sequences in total after excluding sequences overlapping between persons, and 79 sequences of them existed significantly (*P* = 0.0000097–0.0498) more or less abundantly in plasma from the myeloma patients than in that from the healthy persons. Representative 6 comparisons are shown in Fig. 3 and the others are in S3 Table. The 79 sequences included those of fragments of Y-RNA, 28S rRNA, and miRNA, and the numbers of the sequences that were more and less abundant in the myeloma patients were 40 and 39, respectively. In the representatives, a 15-nt NACC2 mRNA fragment and a 31-nt Y4-RNA-related fragment were significantly (*P* = 0.0032 and 0.0020, respectively) more abundant in the myeloma patients, while a 10-nt unidentified RNA fragment, a 20-nt 28S rRNA fragment, a 21-nt let-7b miRNA, and a 34-nt Y5-RNA fragment were significantly (*P* = 0.0013–0.0036) less abundant in the myeloma patients (Fig. 3). Among the 753 comparing sequences, only 54 were significantly (*P* = 0.00061–0.0498) more or less abundant in groups of three persons containing one or two myeloma patients, while 79 were so in the myeloma patients. Given random distributions, an expected value of the numbers of the sequences that were significantly more or less abundant in the myeloma patients is 13.3 (= [79 + 54] × 1/_6_ *C*_3_/2) and the probability of that happening is 2.4 × 10^-43^ in the binomial test, suggesting that the myeloma patients are significantly segregated from the healthy persons with respect to small plasma RNA abundance.

However, since the average age (69 years) of the myeloma patients we examined was older than that (27 years) of the healthy persons (S1 Table), those differences in the small RNA abundance may partly reflect the age difference, which is also interesting. It is also possible that the differences in the barcode sequence for reverse transcription (RT)-PCR partly affect the differences in the small RNA frequency among the examined persons. We realize that, due to the small sample size in addition to these, we cannot judge generality of the differences between the myeloma patients and the healthy persons from this study only.

### The 31-nt Y4-RNA fragment

**Fig 4:**
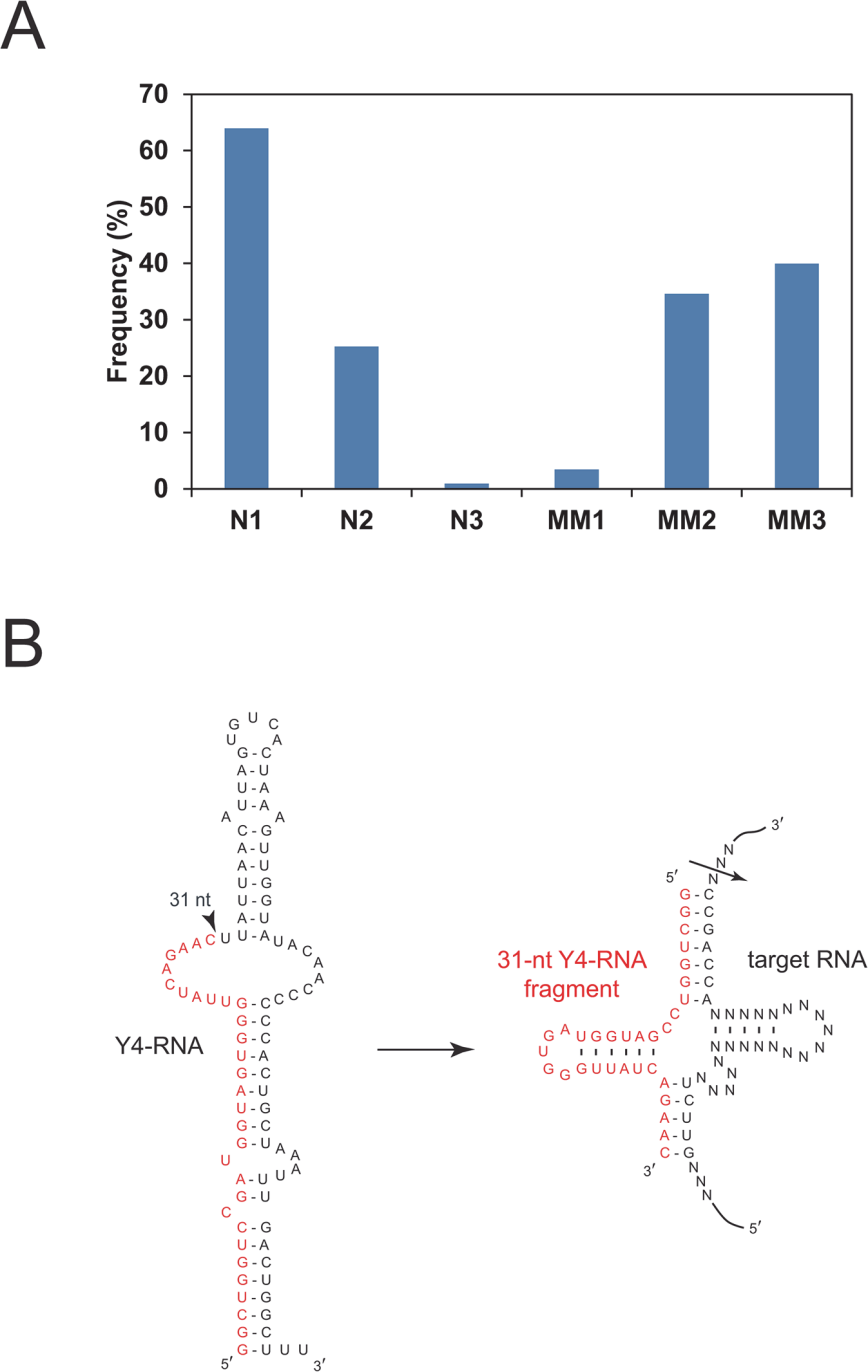
The 31-nt Y4-RNA fragment. (A) The amount of the 31-nt Y4-RNA fragment in plasma. A percentage of a read number of the 31-nt Y4-RNA fragment to a total read number of the 5–40-nt RNAs in plasma are graphed for three healthy persons (N1–N3) and three myeloma patients (MM1–MM3). (B) The structures of human 94-nt Y4-RNA and its 31-nt fragment. The Y4-RNA fragment can form a 5′-half-tRNA-like structure and bind a target RNA to form a pre-tRNA-like structure. An arrowhead and an arrow denote a cleavage site to generate the 31-nt fragment and a potential cleavage site by tRNase Z^L^, respectively.

Two 31-nt Y4-RNA-related fragments with a C-to-A substitution at the 31st or 26th nucleotide were significantly (*P* = 0.0020 and 0.0013) more abundant in the myeloma patients (Fig. 3 and S3 Table). The former fragment may originate from a Y4-RNA pseudogene (RNY4P20) on chromosome 6, and the origin of the latter is unknown. The third 31-nt Y4-RNA-related fragment, the sequence of which matches perfectly the 5′ sequence of 94-nt Y4-RNA, was neither more nor less abundant in the patients, but existed overwhelmingly in a large amount in the plasma of four persons out of six (Fig. 4A). The abundance of 31- and 32-nt Y4-RNA 5′ fragments in human serum and plasma has been reported independently to us [44].

Four types of Y-RNA, Y1-RNA, Y3-RNA, Y4-RNA, and Y5-RNA, are known in human, and each can form a ribonucleoprotein complex with the Ro protein, which is one of the targets of autoantibodies from patients with systemic lupus erythematosus [45,46]. It has been shown that Y-RNAs are involved in the initiation of chromosomal DNA replication and RNA quality control [47,48]. Although emerging of Y-RNA fragments in human Jurkat cells during apoptosis has been also shown, a physiological role of this phenomenon is not clear [49].

We became aware that the 31-nt Y4-RNA 5′ fragment can form a 5′-half-tRNA-like structure to function as an sgRNA for tRNase Z^L^ (Fig. 4B). To test this, we designed and synthesized a 46-nt model RNA target, RNA-Y, which can form a pre-tRNA-like structure with the Y4-RNA fragment (S1A Fig.). The target RNA-Y was examined in the presence of the Y4-RNA fragment for specific cleavage by recombinant human tRNase Z^L^, and it was indeed cleaved at the expected site (S1B Fig.). Together with this property, its abundance in plasma and its presence in the cells (data not shown) would imply its important physiological role as a signaling molecule. The two 31-nt Y4-RNA-related fragments, which can also form 5′-half-tRNA-like structures, would also function as sgRNAs. These observations would warrant further study to find cellular target RNAs of the Y4-RNA fragment and the Y4-RNA-related fragments.

### Small RNA from human PBMC

**Fig 5:**
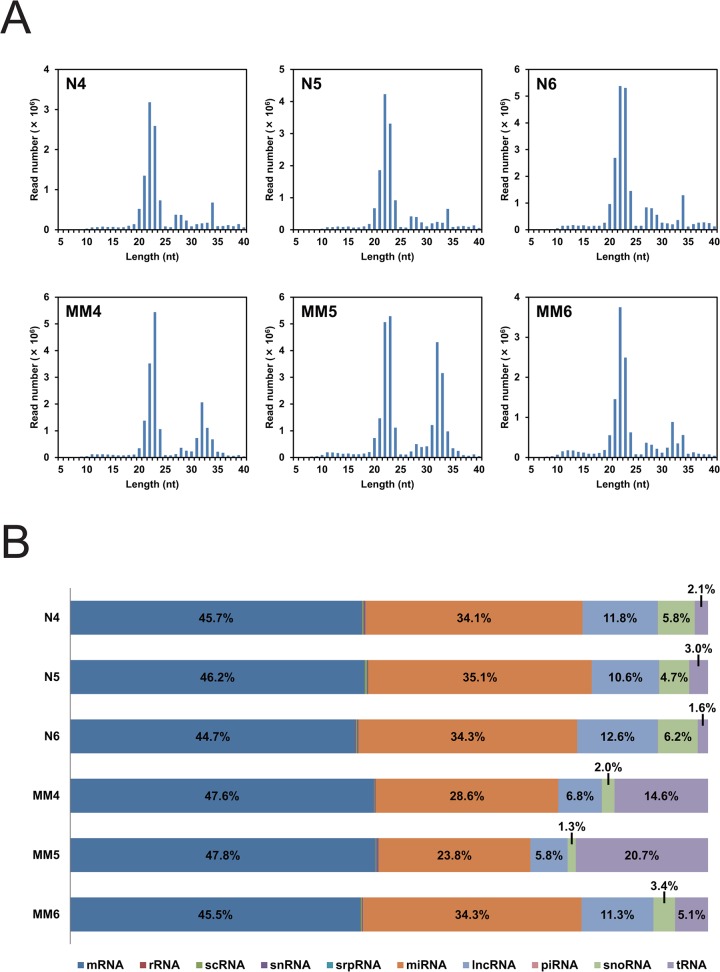
Deep sequencing analysis of small RNA from human PBMC. (A) Frequencies of 5–40-nt RNAs. Read numbers of 5–40-nt RNAs in PBMC from three healthy persons (N4–N6) and three myeloma patients (MM4–MM6) are presented. (B) Relative frequencies of the 10 RNA categories. Each read RNA sequence was assigned to the categories mRNA, rRNA, scRNA, snRNA, srpRNA, miRNA, lncRNA, piRNA, snoRNA, and/or tRNA, and their relative frequencies are graphed for the above six persons.

In the same fashion as above, we analyzed small RNAs from PBMC of three healthy persons (N4–N6) and three multiple myeloma patients (MM4–MM6) for their frequencies and sequences. Although, on the whole, frequency distribution patterns were similar among the six persons, each person showed his/her own distribution pattern (Fig. 5A). The distributions from the healthy persons had peaks at 13, 22, 27/28, and 34 nt in common, while those from the two myeloma patients MM4 and MM5 had peaks at 11/12, 23, 28, and 32 nt in common. The distribution from the myeloma patient MM6 showed a mixed pattern and had peaks at 12/13, 22, 27, 32, and 34 nt. The frequency distribution patterns of the small RNAs from the PBMC were clearly different and easily distinguishable from those from the plasma (Fig. 2A and 5A).

Each read RNA sequence from the PBMC was assigned to its potential original transcript(s), and relative frequencies of the 10 RNA categories are shown for each person (Fig. 5B). The 6 persons had their unique relative frequency distributions, and the categories mRNA, miRNA, lncRNA, snoRNA, and tRNA occupy more than 98% in common. The category scRNA, which occupied a relatively high portion in the frequency distributions of the plasma RNAs, was negligible, while the categories snoRNA and tRNA, which occupied only small portions in the plasma RNAs, were relatively abundant. The percentages of the categories mRNA and miRNA were relatively constant and ranged between 45 and 48% and between 24 and 35%, respectively, while those of the categories lncRNA, snoRNA, and tRNA changed largely by 6–13%, 1–6%, and 2–21%, respectively. The percentage of the category snoRNA was significantly lower in the myeloma patients (2.2 ± 1.1 [mean ± s.d.] vs. 5.6 ± 0.8 with *P* = 0.013). Thus, using this value, we might be able to distinguish myeloma persons from healthy persons.

**Fig 6:**
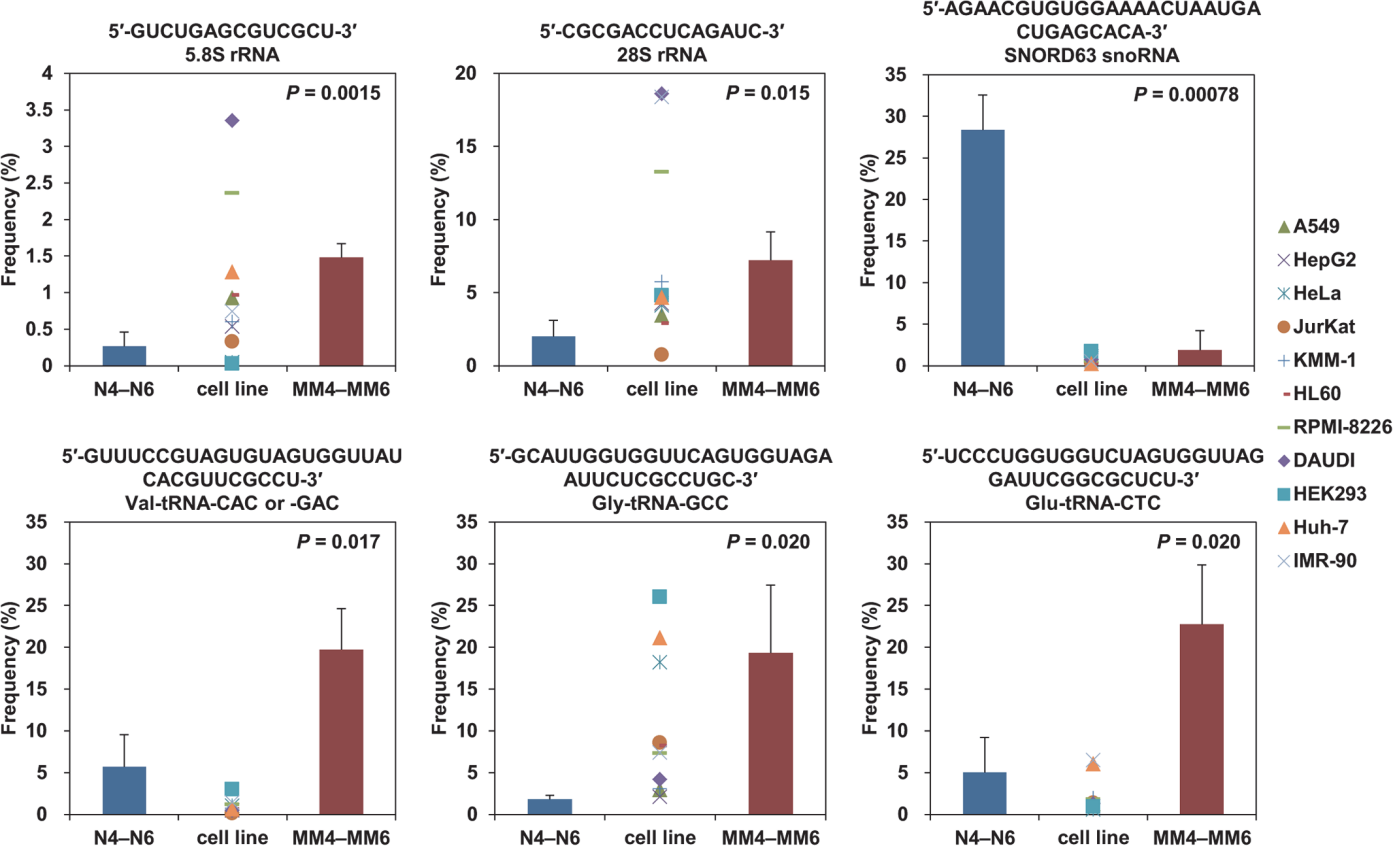
Analysis of RNA species abundant in human PBMC. Average frequencies of each of the six representative RNA species in PBMC from three myeloma patients (MM4–MM6) and in those from three healthy persons (N4–N6) are shown. Their frequencies in the human cell lines A549, HepG2, HeLa, Jurkat, KMM-1, HL60, RPMI-8226, DAUDI, HEK293, Huh-7, and IMR-90 are also plotted. The vertical axis represents the percentage of the read number of each RNA species to the total read number of RNAs with the same nucleotide length. Sequences of the RNA species are presented with their potential original transcript name. Error bars denote standard deviations. These RNA species were significantly more or less abundant in the PBMC from the myeloma patients.

We extracted top 10 abundant RNA sequences for each of 5 to 40 nucleotides in each person and compared frequencies of each RNA sequence among the 6 persons. Among 747 comparing sequences, 109 sequences were significantly (*P* = 0.00011–0.0498) more or less abundant in PBMC from the myeloma patients than in those from the healthy persons. Representative 6 comparisons and the others are shown in Fig. 6 and S4 Table, respectively. For instance, a 14-nt 5.8S rRNA fragment, a 14-nt 28S rRNA fragment, a 33-nt tRNA^Val^ fragment, a 34-nt tRNA^Gly^ fragment, and a 35-nt tRNA^Glu^ fragment were significantly (*P* = 0.0015–0.020) more abundant in the myeloma patients and a 31-nt snoRNA fragment was significantly (*P* = 0.00078) less abundant (Fig. 6). Among the 747 comparing sequences, only 141 were significantly (*P* = 0.00014–0.0496) more or less abundant in groups of three persons containing one or two myeloma patients, while 109 were so in the group of the three myeloma patients. Given random distributions, an expected value of the numbers of the sequences that were significantly more or less abundant in the myeloma patients is 25.0 (= [109 + 141] × 1/_6_ *C*_3_/2) and the probability of that happening is 4.2 × 10^-43^ in the binomial test, suggesting that the myeloma patients are significantly segregated from the healthy persons with respect to abundance of small RNAs from PBMC.

Curiously, in spite of the presence of only tiny portions of the myeloma cells in the PBMC (S1 Table), the above three aspects of the small RNAs in the myeloma patients were distinguishable from those in the healthy persons. This observation may suggest that humoral factors from the myeloma cells and/or systemic cancerous conditions affect RNA metabolism of PBMC. Alternatively, since the average age (67 years) of the myeloma patients was older than that (28 years) of the healthy persons (S1 Table), this age difference may partly affect the differences in the small RNA abundance. The differences in the barcode sequence for RT-PCR may also partly affect the differences in the small RNA frequency among the examinees. We understand that, in addition to these factors, the small sample size makes it difficult to judge generality of the differences between the myeloma patients and the healthy persons.

### 5′-half-tRNAs

We have found a 35-nt 5′-half-tRNA^Glu^ in HEK293 cells, and shown that it can function as a guide RNA for tRNase Z^L^ to cleave a target RNA *in vitro* and that the cellular PPM1F mRNA appears to be one of the targets of 5′-half-tRNA^Glu^-guided tRNase Z^L^ [9]. In this study, we confirmed the existence of the 35-nt 5′-half-tRNA^Glu^ in PBMC, and found that both this 35-nt tRNA^Glu^ fragment and a 36-nt 5′-half-tRNA^Glu^ are more abundant in the PBMC from the myeloma patients than in those from the healthy persons (Fig. 6 and S4 Table). In addition, 29–31-, 34-, and 35-nt 5′-half-tRNAs^Gly^ and a 33-nt 5′-half-tRNA^Val^ were also more abundant in the PBMC from the myeloma patients. This abundance reflects higher percentages (5–21% vs 2–3%) of the category tRNA in the myeloma patients (Fig. 5B). A different subset of those 5′-half-tRNAs were present in each person’s plasma, but their amounts were, on the whole, very low, reflecting small percentages (0.3–1.3%) of the category tRNA (Fig. 2B).

### 5.8S and 28S rRNA fragments

**Fig 7:**
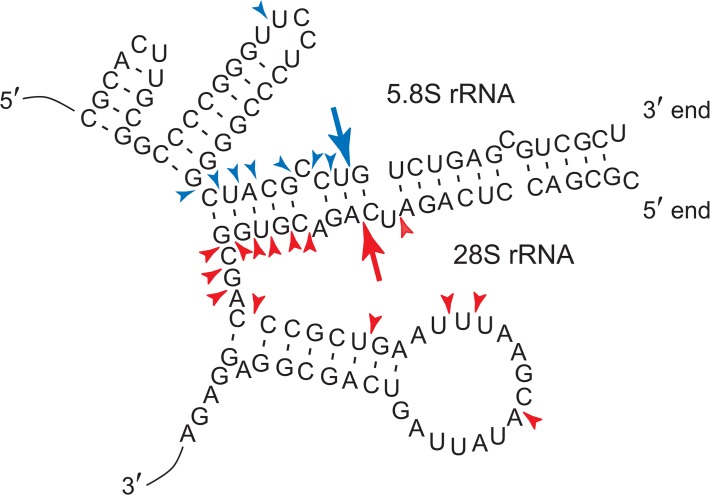
5.8S and 28S rRNA fragments. The 5.8S rRNA fragment 5′-GUCUGAGCGUCGCU-3′ and the 28S rRNA fragment 5′-CGCGACCUCAGAUC-3′ were significantly more abundant in PBMC from the myeloma patients than in those from the healthy persons. Arrows denote the cleavage sites that generate the rRNA fragments. Arrowheads denote cleavage sites that produce various other 5.8S and 28S rRNA fragments in human PBMC.

As mentioned above, the 14-nt 5.8S rRNA fragment 5′-GUCUGAGCGUCGCU-3′ and the 14-nt 28S rRNA fragment 5′-CGCGACCUCAGAUC-3′ were present significantly (*P* = 0.0015 and 0.015, respectively) more abundantly in the PBMC from the myeloma patients than in those from the healthy persons (Fig. 6). Interestingly, these fragments originate from the 3′-end sequence of 5.8S rRNA and the 5′-end sequence of 28S rRNA, respectively, and form a part of a stalk by base-pairings in the 60S ribosomal subunit (Fig. 7). The stalk has been shown to be located on the surface of the subunit, but its function remains to be elucidated [50,51]. Various other 5.8S and 28S rRNA fragments that were generated by cleavages at other sites of the stalk and at its nearby sites were also found in the PBMC (Fig. 7). These rRNA origin fragments were likewise present in human cultured cell lines, but barely present in plasma.

Generally, the amount of the 14-nt 28S rRNA fragment in PBMC was ∼5-fold as high as that of the 14-nt 5.8S rRNA fragment. This may suggest that the 28S rRNA fragment has important functions in the cells. One of them may be to guide tRNase Z^L^ as a 14-nt linear sgRNA to degrade specific RNAs.

### RNA from human cultured cell lines

Furthermore, we examined small RNAs from human cultured cell lines, A549, HepG2, HeLa, Jurkat, KMM-1, HL60, RPMI-8226, DAUDI, HEK293, Huh-7, and IMR-90, for their frequencies and sequences. Although the distributions from the 11 cell lines had peaks at 22 or 23, and 31 or 32 nt in common, each cell line showed its unique distribution pattern (S2A Fig.). On the whole, the distribution patterns were similar to those of RNAs from the PBMC (Figs. 5A and S2A).

Relative frequencies of the 10 RNA categories for each cell line are shown in S2B Fig. The percentages of the category mRNA were relatively constant and ranged between 45 and 49%, and those of most of the other categories varied largely depending on the cell lines, resulting in that each relative frequency distribution showed a unique pattern. With respect to the RNA sequences that were significantly (*P* = 0.00011–0.0498) more or less abundant in PBMC from the myeloma patients than in those from the healthy persons, their frequencies in the 11 cell lines are presented in Fig. 6 and S4 Table. These data suggest that each human cell line appears to show a frequency pattern tilted towards neither that of the healthy persons nor the myeloma patients but its intrinsic pattern.

### Expanding layer of the gene regulatory network

Although the patients MM1 and MM6 are the identical person, the frequency distribution pattern, the relative frequencies of the 10 RNA categories, and the abundant RNA sequences for each of 5 to 40 nucleotides differed clearly between the plasma and PBMC RNAs. This suggests that small RNAs in plasma are not secreted in proportion to those in PBMC. It is not clear which tissues secrete the plasma small RNAs and whether primary secreted RNA molecules are further degraded in plasma. We also do not know precise mechanisms of how the small degraded RNA products are generated in the cells.

In any case, the existence in plasma of the various 5′-half-tRNAs, the 31-nt Y4-RNA fragment that can function as a 5′-half-tRNA-like sgRNA, and many other small RNAs that can function as heptamer-type sgRNAs and 14-nt linear sgRNAs suggests that the gene regulatory network via tRNase Z^L^ and sgRNA may be extended intercellularly (Fig. 1). This supposition would be further supported by the observations that human plasma contains various species of 5′-half-tRNA and miRNA [52], a subset of which can work as 5′-half-tRNA-type or hook-type sgRNA [14,53].

### Potential plasma RNA markers

The present analysis for plasma RNAs, albeit small in sample size and age-unmatched, suggests a possibility that frequencies of the specific RNA species may be used for distinguishing myeloma patients from healthy persons (Fig. 3 and S3 Table). To prove this possibility, we are planning to perform plasma RNA analysis for age-matched large cohorts. If proved, our analysis for plasma small RNAs may be also used for an indicator of personal healthiness as well as for diagnosis and prognosis for other types of cancer. Furthermore, if a subset of the plasma small RNAs are indeed used as intercellular signaling molecules for homeostasis and are involved in the pathogenesis of some diseases, some of the RNA molecules or their antagonists can become good candidates of therapeutic agents.

## Supporting Information

S1 Fig The 31-nt Y4-RNA fragment as an sgRNA. (A) A structure of a complex of the Y4-RNA fragment with the model target RNA-Y. An arrow indicates the expected tRNase Z^L^ cleavage site. (B) *In vitro* tRNase Z^L^ cleavage assays. 5′-fluorescein-labeled RNA-Y was incubated for the indicated time periods with recombinant human Δ30 tRNase Z^L^ in the absence or presence of the Y4-RNA fragment. The cleavage products were analyzed on a denaturing 10% polyacrylamide gel.
(PDF)

S2 Fig Deep sequencing analysis of small RNA from human cultured cell lines. (A) Frequencies of 5–40-nt RNAs. Read numbers of 5–40-nt RNAs in the human cultured cell lines A549, HepG2, HeLa, Jurkat, KMM-1, HL60, RPMI-8226, DAUDI, HEK293, Huh-7, and IMR-90 are graphed. (B) Relative frequencies of the 10 RNA categories. Each read RNA sequence was assigned to the categories mRNA, rRNA, scRNA, snRNA, srpRNA, miRNA, lncRNA, piRNA, snoRNA, and/or tRNA, and their relative frequencies are presented for the eleven cell lines.
(PDF)

S1 Table The information on examinees. (XLSX)

S2 Table The information on cDNA libraries. (XLSX)

S3 Table RNA species abundant in human plasma. (XLSX)

S4 Table RNA species abundant in human PBMC. (XLSX)
