# Supplementary figures and images for: Potential Small Guide RNAs for tRNase ZL from Human Plasma, Peripheral Blood Mononuclear Cells, and Cultured Cell Lines
Source: PLoS One. 2015 Mar 2;10(3):e0118631. doi: 10.1371/journal.pone.0118631 (PMC4346264; doi:10.1371/journal.pone.0118631)

A

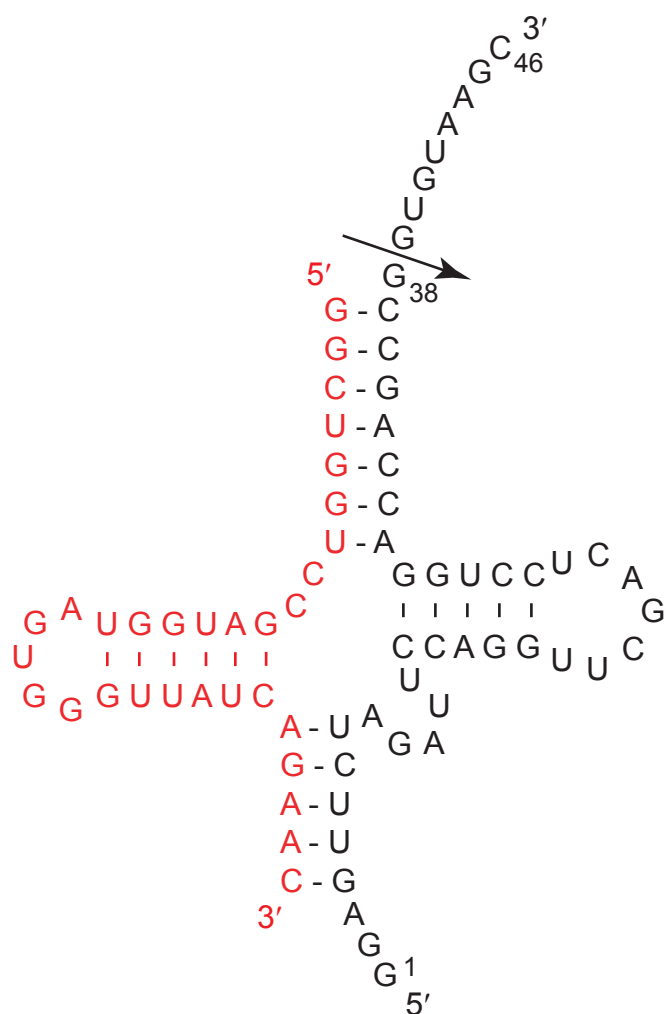

B

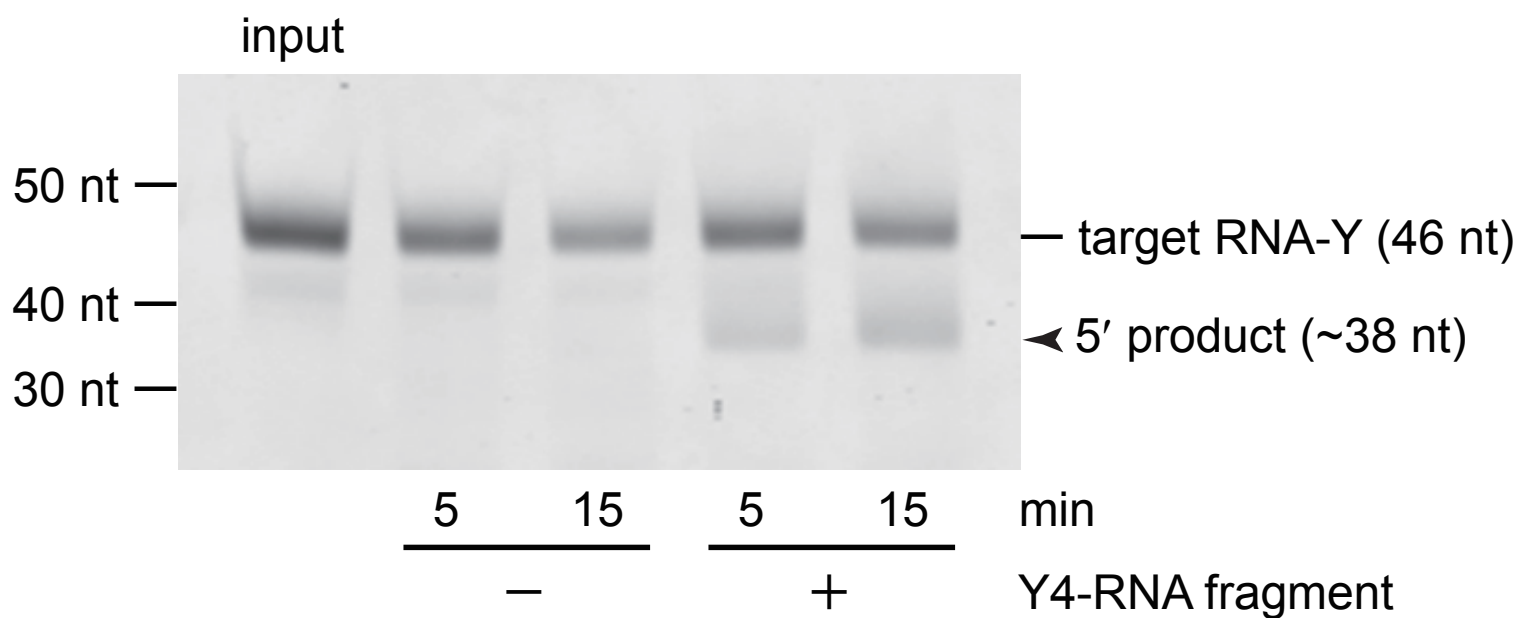

Figure S1

Supplement: S1 Fig — (A) A structure of a complex of the Y4-RNA fragment with the model target RNA-Y. An arrow indicates the expected tRNase ZL cleavage site. (B) In vitro tRNase ZL cleavage assays. 5′-fluorescein-labeled RNA-Y was incubated for the indicated time periods with recombinant human Δ30 tRNase ZL in the absence or presence of the Y4-RNA fragment. The cleavage products were analyzed on a denaturing 10% polyacrylamide gel. (PDF) [file pone.0118631.s001.pdf]

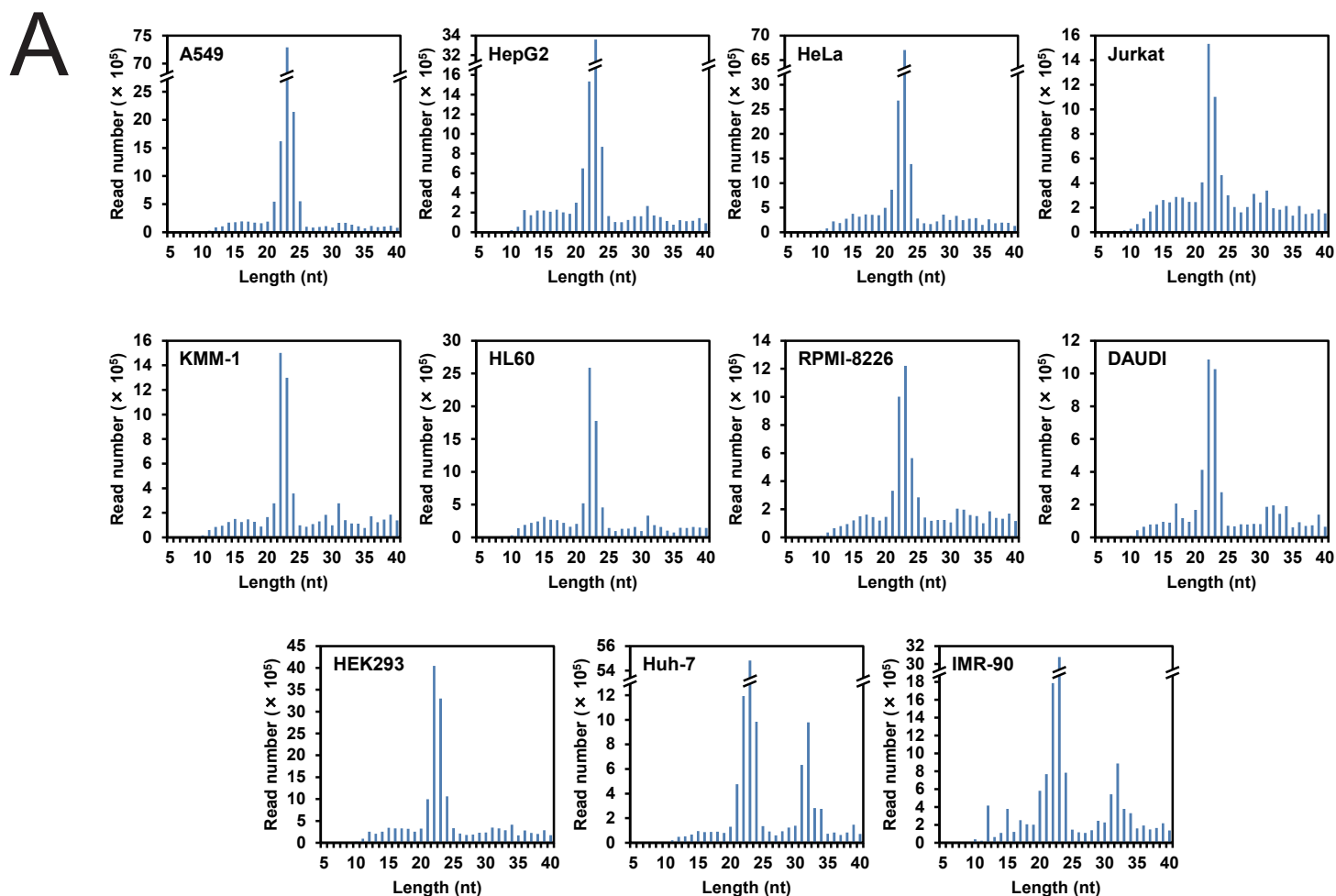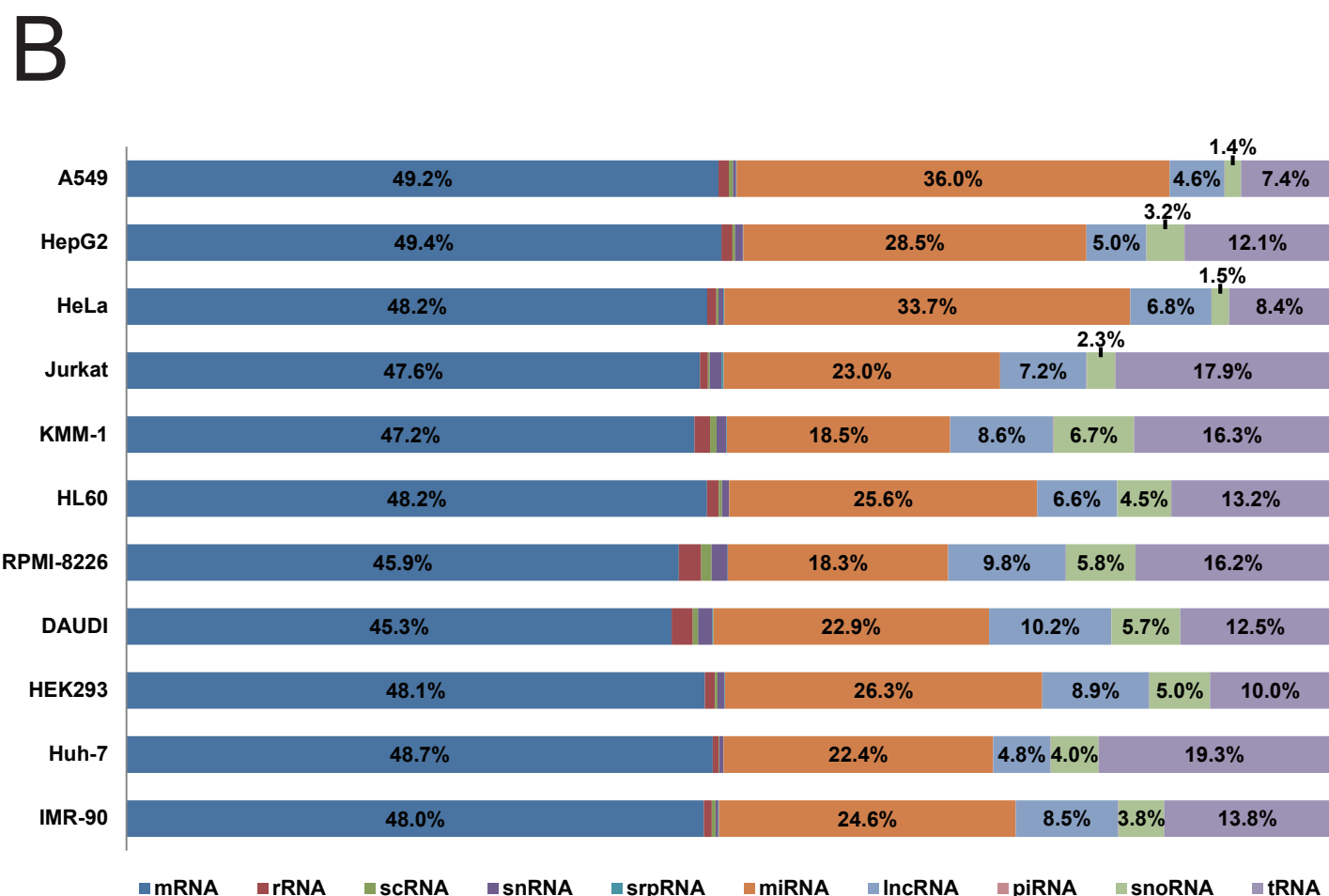

Figure S2

Supplement: S2 Fig — (A) Frequencies of 5–40-nt RNAs. Read numbers of 5–40-nt RNAs in the human cultured cell lines A549, HepG2, HeLa, Jurkat, KMM-1, HL60, RPMI-8226, DAUDI, HEK293, Huh-7, and IMR-90 are graphed. (B) Relative frequencies of the 10 RNA categories. Each read RNA sequence was assigned to the categories mRNA, rRNA, scRNA, snRNA, srpRNA, miRNA, lncRNA, piRNA, snoRNA, and/or tRNA, and their relative frequencies are presented for the eleven cell lines. (PDF) [file pone.0118631.s002.pdf]
